# Genetic mapping of a pollinator preference trait: Nectar volume in sunflower (*Helianthus annuus* L.)

**DOI:** 10.3389/fpls.2022.1056278

**Published:** 2022-12-19

**Authors:** Ashley C. Barstow, Jarrad R. Prasifka, Ziv Attia, Nolan C. Kane, Brent S. Hulke

**Affiliations:** ^1^ Department of Plant Sciences, North Dakota State University, Fargo, ND, United States; ^2^ Sunflower and Plant Biology Research Unit, Edward T. Schafer Agricultural Research Center, United States Department of Agriculture (USDA)-Agricultural Research Service, Fargo, ND, United States; ^3^ Ecology and Evolutionary Biology Department, University of Colorado, Boulder, CO, United States

**Keywords:** sunflower, *Helianthus annuus*, pollinator preference, genetic mapping, genome structural variation

## Abstract

Although high pollinator visitation is crucial to ensure the yields of pollinator-dependent crops, the quantitative trait loci (QTL) controlling nectar volume in sunflower (*Helianthus annuus* L.), a pollinator preference trait, have yet to be identified. To address this, a recombinant inbred line mapping population, derived from lines with contrasting nectar volume, was used to identify loci responsible for the phenotype. As a result, linkage mapping and QTL analysis discovered major loci on chromosomes 2 and 16 that are associated with variation in nectar volume in sunflower. Increased nectar volume is also associated with increased sugars and total energy available per floret. The regions on chromosomes 2 and 16 associated with the nectar phenotype exhibit indications of chromosome structural variation, such that the phenotype is associated with rearrangements affecting regions containing hundreds of genes. Candidate genes underlying QTL on chromosomes 9 and 16 are homologous to genes with nectary function in *Arabidopsis*. These results have implications for sunflower breeding, to enhance pollination efficiency in sunflower, as well as current and future studies on sunflower evolution.

## Introduction

Nectar is offered as a reward to increase pollinator visitation in many angiosperms (Simpson and Neff, 1983). The ratio of various sugars (most often sucrose, fructose, and glucose; Pham-Delegue et al., 1994) and the wide variety of other components, such as vitamins (Griebel and Hess, 1940), proteins (Carter and Thornburg, 2004) and free fatty acids (Kram et al., 2008), may contribute to pollinator preference (Carter et al., 2006; Raguso and Pichnersky, 1999). However, pollinator behavior has often been considered to approximate optimal foraging, where energy resources (calories) are collected at the lowest cost (time and energy expended) (Pyke, 2016). Because calories available affect bee (*Apis mellifera* L.) growth and development (Burkle and Irwin, 2009), pollinator preference is often associated with the volume (Cnaani et al., 2006) or concentration (Kim et al., 2011) of floral nectar.

Pollinators use a variety of traits to associate which plants previously provided adequate rewards (Knauer and Schiestl, 2015). Pollinators prefer specific flowers from visual and olfactory cues associated with nectar and pollen. For example, gradients of humidity associated with evaporation of nectar can provide reliable information to pollinators regarding nectar rewards (von Arx et al., 2012). The consistency of nectar increases bee fidelity and the efficiency of pollination (Cakmak et al., 2010). Bees will leave a group of plants behind and fly a longer distance when several of the previous reward offerings were low (Pyke, 1978; Thomson et al., 1982; Giurfa and Núñez, 1992; Dukas and Real, 1993; Zimmerman, 1983). Selection for larger volumes of nectar among flowering crops could improve pollination and therefore increase crop production.

Continuous innovation in genetics is principal in sunflower (*Helianthus annuus* L.) breeding to ensure the diversity in breeding populations is keeping up with unforeseeable climate change stresses. Sunflower may become the preferred oil crop in the future as it demonstrates the ability to grow in a broad range of conditions across the world (Miladinović et al., 2019). However, as patterns show a decline in wild pollinators across North America (Cameron et al., 2011) and a forecasted increase in demand for sunflower oil, it is crucial to ensure pollinator visitation is maximized. The most potential to enhance pollination is likely in the breeding programs that target improving reward production. Recent observations of bee visitation and plant traits on released USDA inbred lines show that sunflowers with more nectar and shorter corollas (i.e., easier physical access to nectar) were associated with more bee visits (Mallinger and Prasifka, 2017).

Sunflowers are known to be attractive for both wild and managed pollinators (United States Department of Agriculture [USDA], 2015). Despite the fact that commercial sunflower hybrids are able to self-pollinate, bees generally improve seed yields (Degrandi-Hoffman and Chambers, 2006). Modern sunflower planting seed production relies on a hybrid system that employs cytoplasmic male sterility, which is completely reliant on bees to move pollen from male-fertile to male-sterile plants (Greenleaf and Kremen, 2006). Since nectar volume can significantly affect the frequency of pollinator visitation, knowledge of genes controlling the nectar volume production in sunflowers may be used in the future for selection purposes to ensure optimal yields.

## Materials and methods

### Plant materials

Parental lines to create the mapping population were selected using data on nectar-related traits, specifically sharply contrasting nectar volume and sucrose content (Mallinger and Prasifka, 2017). HA 434 is an oilseed sunflower maintainer line with high oleic acid content in the seed oil (Miller et al., 2004), with a high nectar content and low sugar concentration. HA 456 is also a sunflower maintainer line with high oleic acid in the seed oil, which was derived from a cross of HA 434 with S-16 YU (Miller et al., 2006), and has a low nectar content and high sugar concentration. Despite the co-ancestry of the two lines, they had the most divergent nectar phenotypes found in an observation panel. Because these plants are identical at many sites on the genome due to co-ancestry, this parent choice allowed us to eliminate a large portion of the sunflower genome as contributing causal variation.

The population was founded from a single F_1_ individual from the HA 456/HA 434 cross. The population underwent single seed descent each filial generation until F_6_ seeds were produced. Single seed descent occurred in greenhouses and field environments in Moorhead, MN, USA and Rancagua, Chile in 2017 and 2018.

### Controlled-environment phenotyping

During 2018-2019, 198 entries from the HA 456/HA 434 population were grown. The F_6_ seeds were planted into conical containers (D40 Deepots, Stuewe & Sons Inc., Tangent, OR, U.S.A.) that had been filled with standard soilless growing medium (Pro-Mix B, Premier Tech Horticulture, Quakertown, PA, U.S.A.). The conical containers were kept in the plant growth chamber with 14:10 L:D cycle, constant 28°C temperature and 65% RH. After emergence, seedlings were given 2 g of a controlled release fertilizer (14-14-16 N-P-K; Haifa North America, Savanna, GA, U.S.A.). A water-soluble fertilizer (20-20-20 N-P-K; JR Peters, Inc, Allentown, PA, U.S.A.), mixed to 250 ppm N, was applied one day each week.

The size of the population required plants to be grown in six blocks planted 30-50 days apart. Each block comprised the parents (HA 434, HA 456) and 30–36 entries. Flowering of an entry was considered to have begun (day 1) when all of the outermost florets on the capitulum had started shedding pollen. Over the next three days (days 2-4), the newest pistillate florets were sampled to collect data on nectar volume (µl/floret) and concentration (°Brix). Nectar was collected using a 1 µl microcapillary tube (Drummond Scientific Company, Broomall, PA, U.S.A.). The millimeters of nectar are directly proportional to the microliters in the microcapillary tube. Nectar from three florets was pooled in a single microcapillary tube on each of the three days of sampling with most entries. However, on some of the entries with the most nectar, a single floret was sufficient to use. After the height of nectar in the capillary and number of florets were noted, a bulb was used to dispense nectar onto a small hand-held analog refractometer (Bellingham + Stanley, Royal Tunbridge Wells, UK). For about one in ten entries (21/198), the volume of nectar collected was insufficient for a Brix reading. Because individuals in the mapping population were sampled repeatedly, nectar volume data were averaged over the three successive days. Entries in the population yielded 3–117% the volume of nectar collected from HA 434.

### In-field validation of nectar phenotypes

Parental types and entries with per-floret nectar volumes in the range of values observed for either the high parent (n=10) or low parent (n=9) in the growth chambers were grown and evaluated under field conditions. Each entry was planted in two single-row plots (up to 20 plants in plots 5.2 m long, 0.76 m between rows) on 14 June 2019 at the North Dakota State University Agronomy Seed Farm near Casselton, North Dakota, U.S.A. Plants to be sampled were covered with cloth bags and tied shut to exclude pollinators 24 h prior to nectar sampling. Sampling of plants for nectar volume and concentration (°Brix) used methods as described previously, except using larger (6.66 µl) microcapillary tubes and up to 10 florets per head. Nectar sampling was conducted between 21–30 August, and sampling for each entry included three plants sampled over two consecutive days (different plants each day). Nectar collection from all plants was sufficient for a Brix reading, allowing groups to also be compared on a total sugar basis (nectar volume × concentration). T-tests were calculated to compare the high and low nectar phenotypic groups and Pearson correlations were calculated to assess the relationship between nectar volume and sugar concentration using SAS v. 9.4 (SAS Institute, 2016).

### Genotyping and SNP calling

Lyophilized leaf material for the 192 RILs and the two parental lines were ground using tungsten carbide bearings in a Qiagen 96-well plate shaker (Qiagen, Hilden, Germany). Genomic DNA (gDNA) was extracted from leaf tissues using a Qiagen DNeasy 96 plant kit. The manufacturer’s protocol was modified to include the addition of 10 mM SMB (sodium metabisulfite) to the initial lysis buffer, a 45 minute incubation at 65°C for the ground material in lysis buffer, a 100% ethanol wash before final drying of the membrane prior to elution, and DNA storage in an elution buffer that contained 10 mM DTT (dithiothreitol), which have all been shown to improve DNA concentration and purity (Gao et al., 2018; Pogoda et al., 2018). Extracted samples were stored at -20°C prior to library preparation.

Genomic libraries were prepared following standard protocols using Nextera^®^ XT DNA library prep kits (Illumina^®^) and were barcoded with the Nextera^®^ adaptors i5 and i7. Insert size was 450 bp. Pools that passed QC were processed for an average coverage depth of 5x, 151 bp average read length, paired-end HiSeq^®^ 2000 reads at the Novogene sequencing facility in Sacramento, CA.

Demultiplexed data was downloaded directly from Novogene’s servers. FASTQ data were trimmed using Trimmomatic Version 0.38 (Bolger et al., 2014) with the following parameters: NexteraPE-PE.fa:2:30:10 LEADING:3 TRAILING:3 MINLEN:100, with NexteraPE-PE.fa containing the standard set of Nextera adapters to be trimmed from reads. Resulting FASTQ files were aligned to the most up-to-date assembly available as of writing, HA412-HO.v2.fasta (Badouin et al., 2017). Variant calling was performed using GATK best practices (Van der Auwera et al., 2013; Van der Auwera & O’Connor, 2020). A single variant call file (vcf) table was created.

The vcf table was filtered for single copy sites based on depth (sites with depth across all sunflower lines between 200-1000 were retained). Additionally, filtering was done to select sites with a minimum quality score of 100 (minQ=100), minor allele frequency of 0.05 or greater (maf=0.05) and max missingness value of 0.75 (max-missing=0.75). Missing data were imputed using BEAGLE version 5.0 with default settings retained (Browning, Zhou, and Browning 2018). Resulting SNPs were compared to the parental genotypes to determine which are polymorphic. Polymorphic SNPs were then filtered using a custom script invoking PROC FREQ of SAS v. 9.4 (SAS Institute, 2016) to exclude markers that did not fit the expected F_6_ segregation ratio, 93:6:93, from a Chi-square analysis goodness of fit test (p > 0.10).

### Linkage map construction

JMP^®^, Version 9.0 (SAS Institute, Cary, NC) was used to construct the linkage map with the retained markers. Interactive hierarchical clustering was performed, and the markers were ordered using the Kosambi mapping function. Using linkage group order, the chromosomal locations were brought into the model.

The Linkage Map order was used to determine the optimal order of the markers within the linkage groups and determine the genetic distances from previous recombination frequencies. The Nearby Marker Recombination Constraint was increased to 0.7, to account for the large distances between markers on certain chromosomes. The linkage map was sorted by linkage groups and reordered in preparation for QTL analysis. The QTL genotype probability data set was built using the cross type ‘RI1’ and annotated using the chromosome numbers and Kosambi marker position in centimorgans.

### Identification and validation of QTLs

To identify QTL, the markers from the final linkage map were incorporated into JMP^®^, Version 9.0 (SAS Institute, Cary, NC). Analyzing phenotypic data deviating from normality can drastically alter QTL mapping efficiency (Yang et al., 2006). Therefore, the phenotypic data of nectar volume from the growth chambers, µl/floret, was transformed using a lambda = 0.25 Box-Cox transformation to improve model fit. A multiple interval mapping method (Kao et al., 1999) was used with Haley-Knott regression (Haley and Knott, 1992). A Forward Search for Main Effects was performed first, and the lowest main effect was removed from the model. LOD score thresholding for entry into the model was 1 and retention was set to 2 using a 1.0 test step. The 1-Dimensional search tool was used to detect additive interaction QTLs. LOD score thresholds for two-way QTL interactions were set to 2 for entry and 3 for retention using the default test step.

For the nectar sugar concentration, QTL mapping was attempted with the same protocol as nectar volume without data transformation.

Multiple linear regression was used to validate QTLs using the aforementioned field data set from 2019 (PROC REG of SAS v. 9.4; SAS Institute, 2016). The phenotypic data of nectar volume (µl/floret) was transformed using a lambda = 0.25 Box-Cox transformation to improve model fit before means were generated. Multiple regression was performed on transformed means.

### Candidate gene analysis

Using the reference genome HA412-HO.v2 (Badouin et al., 2017), gene annotations in the significant QTL regions were identified. Significant regions were defined as any interval of adjacent markers with individual QTL LOD values within 2 LOD score increments of a QTL peak. If no markers were within 5 Mbp of a QTL interval margin, the margin was extended by 5 Mbp for the purposes of searching for annotated genes in sunflower. Previous studies on *Arabidopsis thaliana* have identified several nectar production related genes through functional genomics investigations (Roy et al., 2017). Therefore, to show homology, a comparison of the candidate genes within the regions was performed using a blastx (Altschul et al., 1990) of the sunflower genes against the TAIR 11 *Arabidopsis* proteins. A minimum threshold bitscore of 20 was used to include the broadest selection of possible hits.

## Results

### Controlled-environment phenotyping

**Figure 1:**
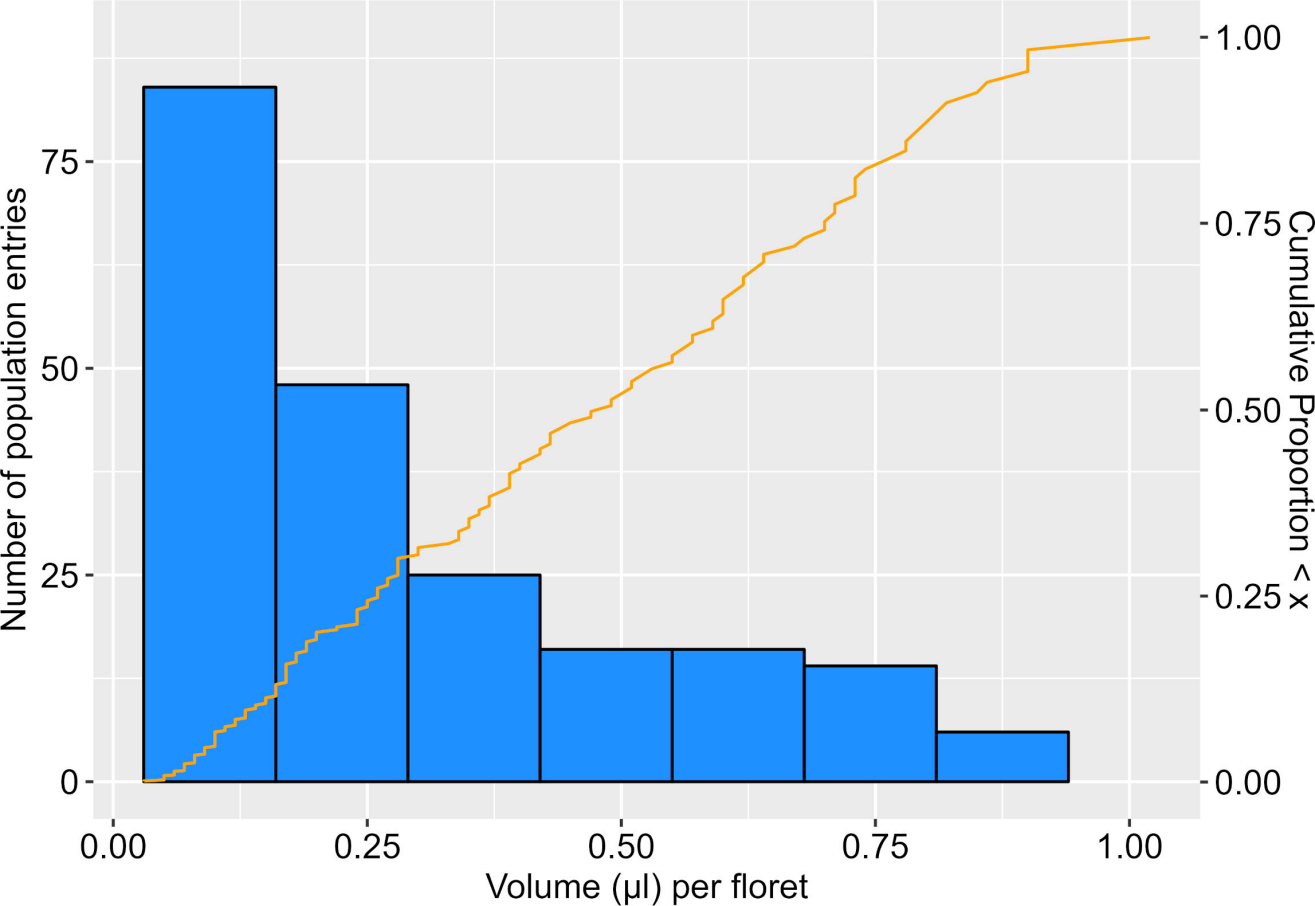
Frequency distribution of nectar volume (µl/floret) in the F6 mapping population. Distribution includes values of the parental types, HA 456 (0.09 µl/floret) and HA 434 (0.83 µl/floret), averaged from repeated sampling across blocks (n=6) used to evaluate population.

Across the six blocks in which the population was evaluated, nectar collections from parental types showed variation, but the high parent, HA 434, showed consistently greater nectar volume (range 0.67-1.02 µL/floret) than the low parent, HA 456 (0.05-0.15 µL/floret). The 198 population entries sampled exhibited nectar volumes similar to the parents, but also with values that appeared intermediate (**Figure 1**). In the 176 lines that produced sufficient nectar for concentration (°Brix) measurement, there was a strong negative correlation between nectar volume and concentration (r = -0.732, n=178, P < 0.001).

### Nectar phenotype in field validation

**Figure 2:**
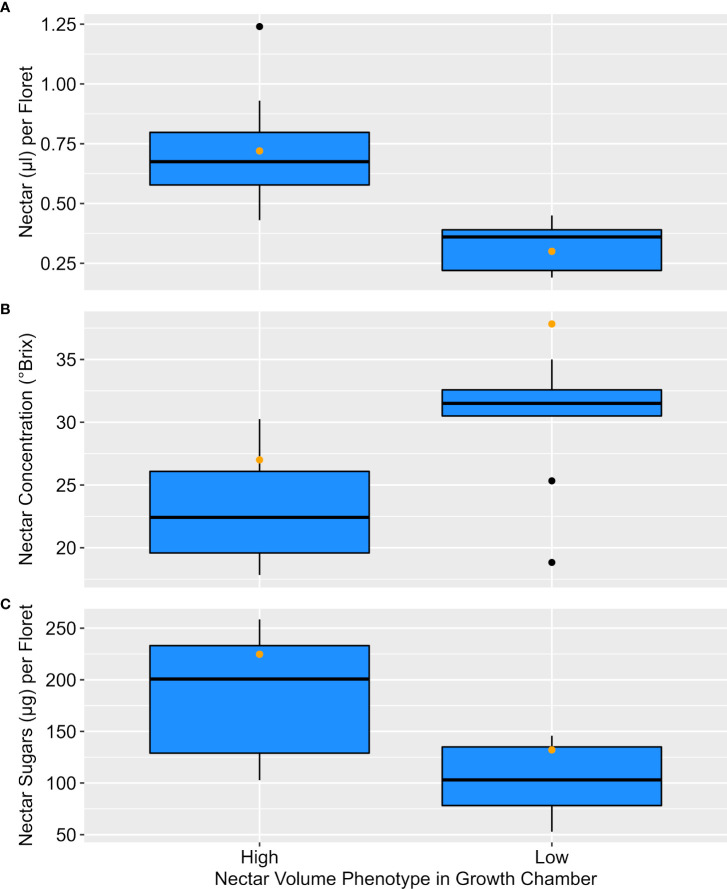
Nectar phenotypes under field conditions for entries assessed as similar to the low parent (n=9) or parent (n=10) in controlled-environment phenotyping. High and low groups differed (t-test P < 0.05) for **(A)** nectar volume (µl/floret), **(B)** nectar concentration (°Brix), and **(C)** sugar (µg/floret). Orange dots denote the high and low parent phenotypes.

Groups of entries with per-floret nectar volumes similar to the high parent (n=10) or low parent (n=9) in growth chambers were also found to differ for nectar concentration (°Brix), and total sugar (µg/floret) under field conditions. On average, the high nectar entries produced over twice the nectar volume (t = 4.92, df = 17, P < 0.001) as low nectar entries, but also nectars that were about a quarter less concentrated (i.e., lower °Brix; t = -3.24, df = 17, P = 0.005). When both nectar volume and concentration are combined to estimate sugar (µg/floret), high nectar entries provided about 75% more sugar (t = 3.57, df = 17, P = 0.002). Distribution of values for entries in each group are shown in **Figure 2**. Interestingly, the distribution of both subgroups suggests the potential for transgressive segregation in the nectar volume and total sugar phenotypes. As with controlled-environment phenotyping, nectar volume and concentration were negatively correlated in the field (r = -0.537, n = 19, P = 0.018).

### Construction of the linkage map

**Figure 3:**
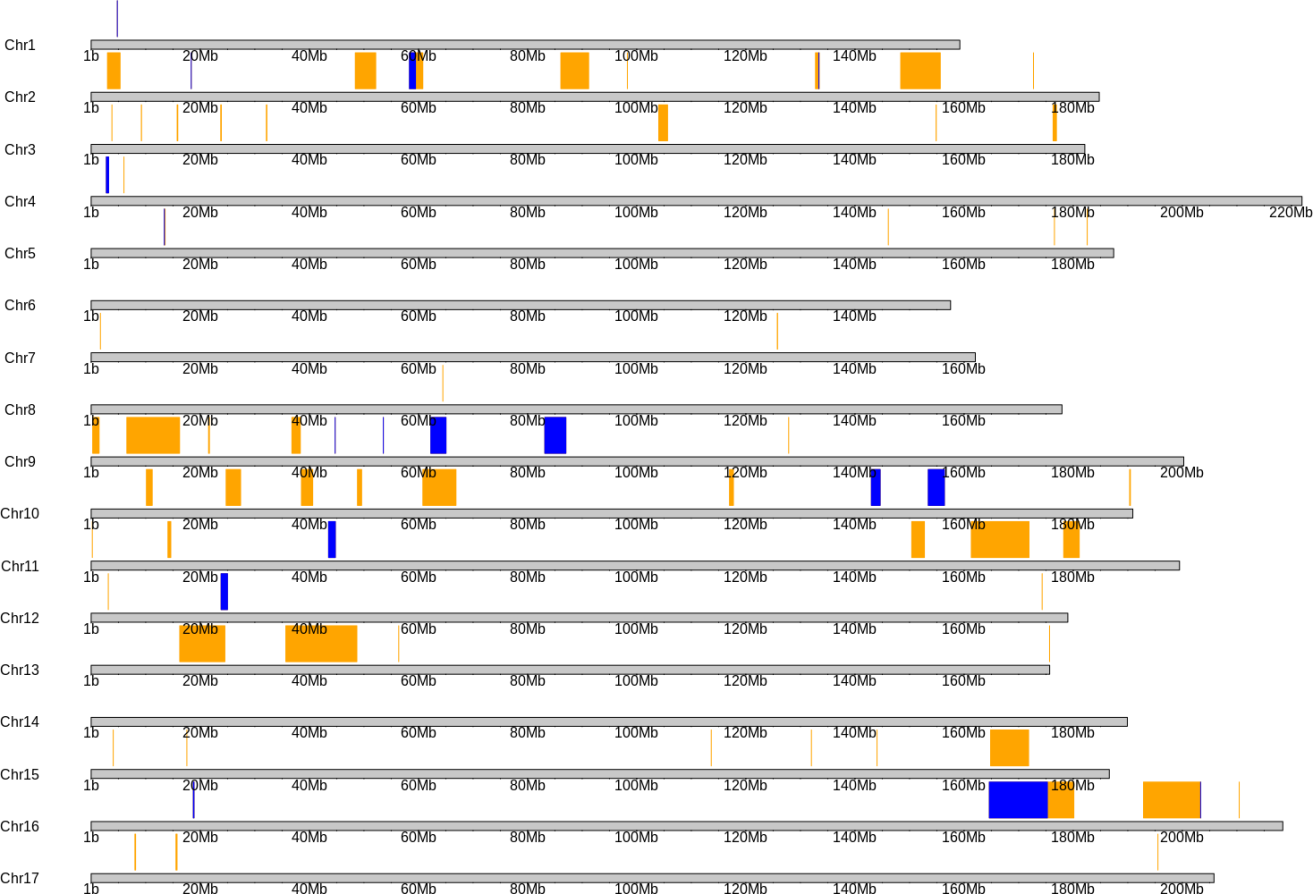
Physical map of the sunflower genome, as aligned to the HA 412HOv2 genome. Regions of chromosomes with orange or blue boxes highlighting above the karyotype indicates regions polymorphic between HA 456 and HA 434. The remaining regions are either monomorphic due to identity by descent or are in repetitive regions, which were filtered out during the variant calling process. Blue boxes indicate nectar volume quantitative trait loci (within 2 LOD units of peak significance).

The linkage map contained 764 markers spanning across fifteen different chromosomes, excluding chromosomes 6 and 14 (**Figure 3**). For the 15 chromosomes, the number of markers ranged from 2-125 per chromosome, and formed localized clusters on each, due to the identity by descent of chromosome segments from HA 434 to HA 456. Chromosome 6 only contained one marker and was excluded from the analysis, as it was likely a genotyping error. Since the parents of the biparental population were inbred lines and both were primarily selected for their high oleic oil type, chromosome 14 appears to have been transferred from HA 434 to HA 456 intact, thus resulting in no polymorphic markers. Chromosome 14 contains the *Fad2-1* locus, which is a gene of large effect for oleic acid content (Schuppert et al., 2006).

### QTL analysis of nectar volume and sugar concentration

**Figure 4:**
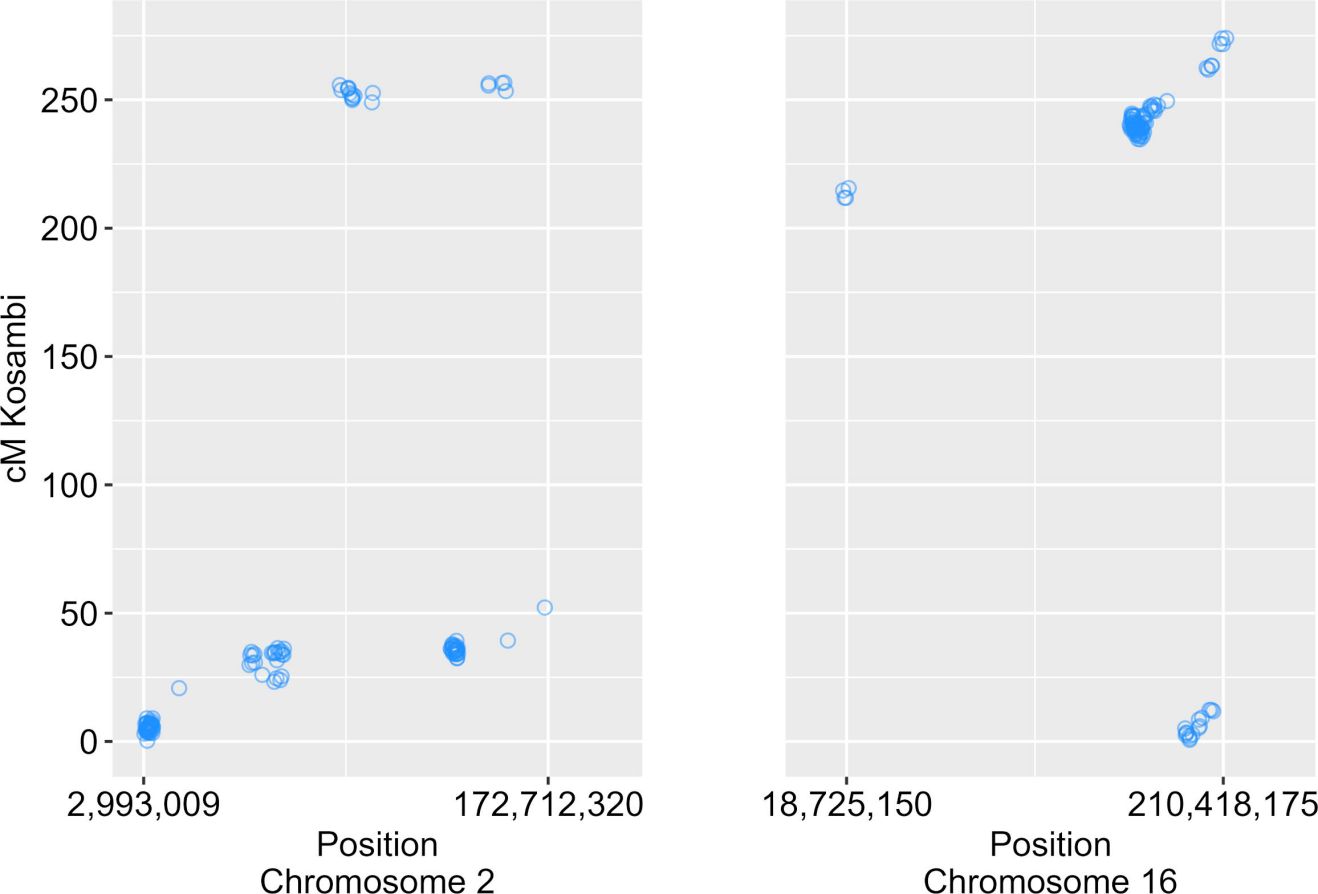
Scatterplot of physical positions of markers on chromosomes 2 and 16 versus genetic map positions. Inverse diagonal trends indicate the presence of inverted sequence relative to the reference genome HA 412HOv2. Off diagonal trends indicate translocated sequences.

We identified 12 QTLs associated with nectar volume mapped across chromosomes 1, 2, 4, 5, 9, 10, 11, 12 and 16 (**Table 1**; **Figure 3**). The LOD scores for the main effects and epistatic interactions range from 2.5 to 10.7 with the highest mapping on chromosome 5. After stepwise regression upon the analysis of a 1-D scan, the results showed two QTLs with significant main effects detected on chromosome 2 and two more on chromosome 16. On both chromosomes, the two QTL were nearby but not closely linked, and had opposing effects relative to the parental haplotypes. On chromosome 2, there appears to be an inverted sequence when compared to the reference genome, HA412HO. Additionally, chromosome 16 appears to have two translocations (relative to HA 412HO; **Figure 4**). Significant interaction effects were found between chromosomes 1 and 5, 4 and 12, 5 and 12, 10 and 16, 11 and 16, and two loci on 16. The effect estimates of the epistatic effects were smaller than the main effects of the loci on chromosomes 2 and 16.

**Table 1:** Summary of QTL effects on the nectar volume trait in sunflower floral nectaries.

QTL1 start	QTL1 end	Peak LOD	QTL2 start	QTL2 end	Peak LOD	Effect estimate^a^
Main effects						
Chr2_18340954	Chr2_59449965	2.46				-0.25
Chr2_133380780	Chr2_133381066	3.56				0.29
Chr16_164616872	Chr16_175260575	6.13				-0.27
Chr16_203348602	Chr16_203348909	3.60				0.22
Interaction effects						
Chr1_4794574	Chr1_4794940	4.45	Chr5_13410246	Chr5_13417212	10.68	-0.14
Chr4_2776813	Chr4_3207363	4.31	Chr12_23815559	Chr12_24993888	7.98	0.13
Chr5_13410246	Chr5_13417212	10.68	Chr9_44718755	Chr9_87006964	3.63	0.15
Chr5_13410246	Chr5_13417212	10.68	Chr12_23815559	Chr12_24993888	7.98	-0.18
Chr10_142996839	Chr10_156386987	2.98	Chr16_164616872	Chr16_175260575	8.12	0.12
Chr11_43514256	Chr11_44755672	4.24	Chr16_18725150	Chr16_18847793	7.54	-0.14
Chr16_18725150	Chr16_18847793	7.54	Chr16_203348602	Chr16_203348909	7.20	-0.16

a Positive values indicate that the high parent (HA 434) allele increases the nectar content per floret.

When QTL analysis was attempted with the sugar concentration phenotype, no significant QTL were found. With a very negative and significant correlation between volume and sugar concentration, the sugar concentration data were right-censored because we were unable to generate a large enough sample of nectar to test sugar concentrations for those genotypes with near-zero nectar volume. No significant QTL is likely the effect of the expected lack of statistical power with a tail-censored data set.

### Field validation of nectar volume QTL

The four main effect loci were validated using our field environment dataset from 2019. All four markers were included in a multiple regression model to determine if the markers properly modelled trends in nectar volume. The model was very significant (F=6.37, p=0.003) and explained 63% of the total phenotypic variation.

### Candidate genes

Of the loci identified in the QTL analysis, 15 regions of the genome were analyzed for candidate genes (**Table 2**). The number of identified genes in each region ranged from 0 to 359 with chromosome 9 containing the most genes but also the largest QTL segment length. Several nectary genes in *Arabidopsis* have been previously identified; therefore, these QTL regions were analyzed for homologous genes. Two genes related to nectar production in *Arabidopsis* showed homology when compared to the annotated sunflower genes, *ARF8* located in the QTL region on chromosome 9 and *DAD1* located in the QTL region on chromosome 16. These candidate genes had bitscores of over 100 in our blastx analysis, indicating a very high homology between these two sunflower candidate genes and the genes in *Arabidopsis* with functional genomics support (Roy et al., 2017).

**Table 2:** Candidate genes discovered within nectar QTL intervals on the HA 412HO.v2 sunflower genome.

Chromosome	Start bp	End bp	Length	# Genes
Ha412HOChr01	4,794,574	4,794,940	366	1
Ha412HOChr02	18,340,954	18,340,954	0	2
Ha412HOChr02	58,334,204	59,449,965	1,115,761	7
Ha412HOChr02	89,966,637	89,968,483	1,846	0
Ha412HOChr02	133,380,780	133,381,066	286	0
Ha412HOChr04	2,776,813	3,207,363	430,550	9
Ha412HOChr05	13,410,246	13,417,212	6,966	0
Ha412HOChr09	21,523,160	21,523,632	472	0
Ha412HOChr09	44,718,755	87,006,964	42,288,209	379
Ha412HOChr10	142,996,839	156,386,987	13,390,148	202
Ha412HOChr11	43,514,256	44,755,672	1,241,416	25
Ha412HOChr12	23,815,559	24,993,888	1,178,329	28
Ha412HOChr16	18,725,150	18,847,793	122,643	4
Ha412HOChr16	164,616,872	175,260,575	10,643,703	115
Ha412HOChr16	203,348,602	203,348,909	307	1

## Discussion

Floral rewards are critical to recruitment of pollinators for commercial sunflower seed production, due to the requirement to transfer pollen between inbred lines to make hybrid seed, but also have implications for producers who grow the hybrid seed to produce commodity sunflower. As nectar volume has been elucidated as an important variable in pollinator foraging (Mallinger and Prasifka, 2017), knowledge of this trait in sunflowers will be critical to the deployment of pollinator-friendly inbred lines and hybrids to improve efficiency of hybrid seed production and improve yield of commodity sunflower. The volume of nectar produced by sunflower varies markedly among genotypes (Mallinger and Prasifka, 2017), and our study population exemplifies that these differences even occur in closely related genetic backgrounds, indicating that this is a relatively easy target for breeding. Outside of direct effects on sunflower pollination, floral rewards in mass-flowering crops (e.g., sunflower, oilseed rape, blueberry, almond) are important in supporting populations of wild bees (Holzschuh et al., 2013), suggesting that increased nectar rewards could have additional benefits in areas where sunflower is a major crop.

We found the relationship between nectar volume (i.e., general availability of the resource) and concentration are inversely correlated, initially suggesting that high nectar volume genotypes are simply “watering down” the available sugars. However, a closer look at the data shows that the total amount of sugars is also greater in the high nectar genotypes, demonstrating that the energy content per floret (calories) is increased in the high nectar volume genotypes within our population. Nectar concentration is important to pollinators, which have clear preferences with respect to viscosity of the nectar (Kim et al., 2011). However, because of environmental effects on sunflower nectar concentration (Chabert et al., 2020), whether a particular nectar phenotype is preferred by bees (or adaptive to the plant by increasing pollinator visits) may be context-dependent; a nectar that is dilute and less-desirable in a typical sunflower growing environment may be more attractive under arid conditions.

Linkage mapping and associated QTL discovery is the preferred approach to understanding the inheritance of a trait with extensive, heritable variation that is pervasive throughout a high-performing, modern germplasm base. Our QTL mapping based on phenotypes from our growth chamber experiment resulted in two loci on each of two chromosomes (chromosomes 2 and 16) with significant main effects, and these loci explained much of the phenotypic variation in our field validation experiment. Interestingly, the main effects of the proximal but loosely linked loci on each chromosome had opposing effects. While it is not surprising to have some beneficial loci arising from the phenotypically inferior parent, especially given their close genetic relationship, the occurrence of opposing effects in the parental haplotypes is surprising, and perhaps indicative of the potential for genetic improvement in nectar content. Indeed, there were signs of transgressive segregation compared to both the low and high parents for both nectar volume and total sugars (**Figure 2**).

In constructing the linkage map, we discovered interesting cytogenetic features on chromosomes 2 and 16 when comparing the genetic map with the physical map (mapped to the reference genome of HA 412HO – a genotype derived from the same sunflower genetic pool as HA 434 and HA 456; Miller et al., 2004; Miller et al., 2006). Local inversions have previously been reported for linkage groups 2 and 16 in a consensus genetic map (Talukder et al., 2014). Our results also suggest an inversion on chromosome 2 since a fairly large portion of the physical map is inverted on the genetic map. Additionally, two translocated sequences were detected on chromosome 16, which might explain the multiple QTL and the interactions between them. There were three QTL found on chromosome 16, two with significant main effects and the other implicated in epistatic interactions that included or were near the translocated sequences. Taken together with the observation of opposing genetic effects in adjacent haplotypes suggest that these two important regions for nectar volume per floret are affected by cytogenetic processes in chromosome evolution. Recent studies have similarly found variation for adaptive traits associated with regions that are active sites of chromosome evolution, including structural variants such as translocations and inversions. Flowering time and height were influenced by a single, large haplotypic block in *H. argophyllus* (a closely related species to domesticated sunflower) containing structural variants; further, seed size is governed by two large haplotypic blocks in *H. petiolaris* and is known to affect fitness in sand dune versus non-dune environments (Todesco et al., 2020). Ultraviolet light reflecting pigments, associated with temperature and relative humidity clines in wild *H. annuus* (the progenitor species of cultivated sunflower) was associated with large promoter indels, affecting gene expression (Todesco et al., 2022). Because we do not have *de novo* assemblies of HA 434 and HA 456 at this time, we are unable to propose specific mechanisms that could be at play to influence nectar volume, but the presence of genome structural variation in our linkage map and previous studies suggest that nectar related traits are potentially related to multiple co-adapted genes that are important together rather than a single gene of large effect.

Functional characterization of genes affecting nectar secretion in model organisms, specifically *Arabidopsis*, allows us to look for genes in the QTL regions that may have key roles in developing the high and low nectar phenotypes. Candidate genes ARF8 and DAD1 have been shown to be related to nectar secretion in *Arabidopsis.* Auxin (IAA, indole acetic acid) is a key hormone in plant development that regulates nearly all aspects of development as well as stress responses (Browse, 2009; Kazan and Manners, 2011; Kazan and Manners, 2012). ARF8 is an auxin response transcription factor which has implications in regulating nectar production, with a homolog on sunflower chromosome 9 within the boundaries of our most significant interaction QTL. Crosstalk between the jasmonic acid (JA) pathway and auxin response pathways in nectaries has been identified (Schmitt et al., 2018). DAD1 is a jasmonate signaling and response gene identified in *Arabidopsis*, with a homolog present in the major chromosome 16 QTL in sunflower. Both genes could contribute to the difference in phenotype of nectar volume, as previously studies have reported that the crosstalk between JA and auxin is essential for the regulation of nectary function (Schmitt et al., 2018). For example, JA levels in *B. napus* flowers peak prior to anthesis which happens to coincide with nectar production (Radhika et al., 2010). Also, in *Arabidopsis*, IAA acts through ARF6 and ARF8 to induce JA synthesis and leads to the expression of MYB21 and MYB24 which have both been shown to play roles in flower maturation (Nagpal et al., 2005; Reeves et al., 2012). However, despite these being strong candidate loci, other surrounding genes in the candidate regions may be key in affecting nectar production instead of or addition to these most obvious candidate genes.

The results of our paper provide, for the first time, genomic regions with genetic markers that can guide selection of floral nectar in sunflower traits, and links them to genes with known function in nectaries. Although the findings of our study are relevant to sunflower breeders that are interested in enhancing nectar volume and total energy from each floral nectary, further improvements in nectar rewards may be possible by focused study of variation at these loci in diversity panels or through focused breeding efforts.

## Data availability statement

The datasets presented in this study can be found in online repositories. The genomic data presented in this study are deposited in the Short Read Archive repository of NCBI, BioProject number PRJNA907922.

## Author contributions

BH, JP, and NK conceived and organized the project, JP conducted nectar sampling and analysis, BH conducted tissue sampling, NK and ZA developed libraries, provided oversight to sequencing, and developed variant calls. AB and BH filtered the variant calls and conducted trait mapping. AB led drafting of the manuscript. All authors contributed to the article and approved the submitted version.

## References

[B1] Altschul S. F. Gish W. Miller W. Myers E. W. Lipman D. J. (1990). Basic local alignment search tool. J. Mol. Biol. 215, 403–410. doi: 10.1016/S0022-2836(05)80360-2 2231712

[B2] Badouin H. Gouzy J. Grassa C. J. Murat F. Staton S. E. Cottret L.. (2017). The sunflower genome provides insights into oil metabolism, flowering and asterid evolution. Nature 546, 148–152. doi: 10.1038/nature22380 28538728

[B3] Bolger A. M. Lohse M. Usadel B. (2014). Trimmomatic: A flexible trimmer for illumina sequence data. Bioinformatics 30, 2114–2120 doi: 10.1093/bioinformatics/btu170 24695404 PMC4103590

[B4] Burkle L. Irwin R. (2009). Nectar sugar limits larval growth of solitary bees (Hymenoptera: Megachilidae). Environ. Entomol. 38, 1293–1300. doi: 10.1603/022.038.0441 19689912

[B5] Browning B. L. Zhou Y. Browning S. R. (2018). A one-penny imputed genome from next-generation reference panels. Am. J. Hum. Genet. 103, 338–348. doi: 10.1016/j.ajhg.2018.07.015 30100085 PMC6128308

[B6] Browse J. (2009). Jasmonate passes muster: A receptor and targets for the defense hormone. Annu. Rev. Plant Biol. 60, 183–205. doi: 10.1146/annurev.arplant.043008.092007 19025383

[B7] Cakmak I. Song D. S. Mixson T. A. Serrano E. Clement M. L. Savitski A.. (2010). Foraging response of Turkish honey bee subspecies to flower color choices and reward consistency. J. Insect Behav. 23, 100–116. doi: 10.1007/s10905-009-9199-7

[B8] Cameron S. A. Lozier J. D. Strange J. P. Koch J. B. Cordes N. Solter L. F.. (2011). Patterns of widespread decline in north American bumble bees. Proc. Natl. Acad. Sci. U.S.A. 108, 662–667. doi: 10.1073/pnas.1014743108 21199943 PMC3021065

[B9] Carter C. Shafir S. Yehonatan L. Palmer R. G. Thornburg R. (2006). A novel role for proline in plant floral nectars. Naturwissenschaften 93, 72–79. doi: 10.1007/s00114-005-0062-1 16365739

[B10] Carter C. J. Thornburg R. W. (2004). Tobacco nectarin III is a bifunctional enzyme with monodehydroascorbate reductase and carbonic anhydrase activities. Plant Mol. Biol. 54, 415–425. doi: 10.1023/B:PLAN.0000036373.84579.13 15284496

[B11] Chabert S. Sénéchal C. Fougeroux A. Pousse J. Richard F. Nozières E.. (2020). Effect of environmental conditions and genotype on nectar secretion in sunflower (*Helianthus annuus* l.). OCL 27, 51. doi: 10.1051/ocl/2020040

[B12] Cnaani J. Thomson J. D. Papaj D. R. (2006). Flower choice and learning in foraging bumblebees: Effects of variation in nectar volume and concentration. Ethology 112, 278–285. doi: 10.1111/j.1439-0310.2006.01174.x

[B13] Degrandi-Hoffman G. Chambers M. (2006). Effects of honey bee (Hymenoptera: Apidae) foraging on seed set in self-fertile sunflowers (*Helianthus annuus* l.). Environ. Entomol. 35, 1103–1108. doi: 10.1603/0046-225X-35.4.1103

[B14] Dukas R. Real L. A. (1993). Effects of recent experience on foraging decisions by bumble bees. Oecologia 94, 244–246. doi: 10.1007/BF00341323 28314038

[B15] Gao Q.-M. Kane N.C. Hulke B.S. Reinert S. Pogoda C.S. Tittes S. Prasifka J.R. (2018) Genetic Architecture of Capitate Glandular Trichome Density in Florets of Domesticated Sunflower (Helianthus annuus L.) Frontiers in Plant Science 8:2227 10.3389/fpls.2017.02227 PMC5767279 29375602

[B16] Giurfa M. Núñez J. A. (1992). Foraging by honeybees on carduus acanthoides: pattern and efficiency. Ecol. Entomol. 17, 326–330. doi: 10.1111/j.1365-2311.1992.tb01065.x

[B17] Greenleaf S. S. Kremen C. (2006). Wild bees enhance honey bees’ pollination of hybrid sunflower. Proc. Natl. Acad. Sci. U.S.A. 103, 13890–13895. doi: 10.1073/pnas.0600929103 16940358 PMC1564230

[B18] Griebel C. Hess G. (1940). The vitamin c content of flower nectar of certain labiatae. Zeit. Untersuch. Lebensmitt. 79, 168–171. doi: 10.1007/BF01662427

[B19] Haley C. S. Knott S. A. (1992). A simple regression method for mapping quantitative trait loci in line crosses using flanking markers. Heredity 69, 315–324. doi: 10.1038/hdy.1992.131 16718932

[B20] Holzschuh A. Dormann C. F. Tscharntke T. Steffan-Dewenter I. (2013). Mass-flowering crops enhance wild bee abundance. Oecologia 172, 477–484. doi: 10.1007/s00442-012-2515-5 23114428 PMC3655217

[B21] Kao C.-H. Zeng Z.-B. Teasdale R. D. (1999). Multiple interval mapping for quantitative trait loci. Genetics 152, 1203–1216. doi: 10.1093/genetics/152.3.1203 10388834 PMC1460657

[B22] Kazan K. Manners J. M. (2011). The interplay between light and jasmonate signalling during defence and development. J. Exp. Bot. 62, 4087–4100. doi: 10.1093/jxb/err142 21705384

[B23] Kazan K. Manners J. M. (2012). JAZ repressors and the orchestration of phytohormone crosstalk. Trends Plant Sci. 17, 22–31. doi: 10.1016/j.tplants.2011.10.006 22112386

[B24] Kim W. Gilet T. Bush J. W. M. (2011). Optimal concentrations in nectar feeding. Proc. Natl. Acad. Sci. U.S.A. 108, 16618–16621. doi: 10.1073/pnas.1108642108 21949358 PMC3189050

[B25] Knauer A. C. Schiestl F. P. (2015). Bees use honest floral signals as indicators of reward when visiting flowers. Ecol. Lett. 18, 135–143. doi: 10.1111/ele.12386 25491788

[B26] Kram B. W. Bainbridge E. A. Perera M. A. D. N. Carter C. (2008). Identification, cloning and characterization of a GDSL lipase secreted into the nectar of jacaranda mimosifolia. Plant Mol. Biol. 68, 173–183. doi: 10.1007/s11103-008-9361-1 18553138

[B27] Mallinger R. E. Prasifka J. R. (2017). Bee visitation rates to cultivated sunflowers increase with the amount and accessibility of nectar sugars. J. Appl. Entomol. 141, 561–573. doi: 10.1111/jen.12375

[B28] Miladinović D. Hladni N. Radanović A. Jocić S. Cvejić S. (2019). “Sunflower and climate change: Possibilities of adaptation through breeding and genomic selection,” in Genomic designing of climate-smart oilseed crops. Ed. Kole C. (Cham: Springer International Publishing), 173–238. doi: 10.1007/978-3-319-93536-2_4

[B29] Miller J. F. Gulya T. J. Vick B. A. (2004). Registration of two maintainer (HA 434 and HA 435) and three restorer (RHA 436 to RHA 438) high oleic oilseed sunflower germplasms. Crop Sci. 44, 1034–1035. doi: 10.2135/cropsci2004.1034

[B30] Miller J. F. Gulya T. J. Vick B. A. (2006). Registration of three maintainer (HA 456, HA 457, and HA 412 HO) high-oleic oilseed sunflower germplasms. Crop Sci. 46, 2728–2728. doi: 10.2135/cropsci2006.06.0437

[B31] Nagpal P. Ellis C. M. Weber H. Ploense S. E. Barkawi L. S. Guilfoyle T. J.. (2005). Auxin response factors ARF6 and ARF8 promote jasmonic acid production and flower maturation. Development 132, 4107–4118. doi: 10.1242/dev.01955 16107481

[B32] Pham-Delègue M. H. Laloi D. Bailez O. E. (1994). Pollination working group of the national institute of agronomic research (INRA), france. meeting in montfavet, march 8-9, 1994. Apidologie 25, 422–432. doi: 10.1051/apido:19940409

[B33] Pogoda C. S. Keepers K. G. Lendemer J. C. Kane N. C. Tripp E. A. (2018). Reductions in complexity of mitochondrial genomes in lichen-forming fungi shed light on genome architecture of obligate symbioses. Mol. Ecol. 27, 1155–1169. doi: 10.1111/mec.14519 29417658

[B34] Pyke G. H. (2016). Plant–pollinator co-evolution: It’s time to reconnect with optimal foraging theory and evolutionarily stable strategies. Perspect. Plant Ecol. Evol. Syst. 19, 70–76. doi: 10.1016/j.ppees.2016.02.004

[B35] Pyke G. H. (1978). Optimal foraging: Movement patterns of bumblebees between inflorescences. Theor. Population Biol. 13, 72–98. doi: 10.1016/0040-5809(78)90036-9 644520

[B36] Radhika V. Kost C. Boland W. Heil M. (2010). The role of jasmonates in floral nectar secretion. PloS One 5, e9265. doi: 10.1371/journal.pone.0009265 20174464 PMC2824824

[B37] Raguso R. A. Pichersky E. (1999). New perspectives in pollination biology: Floral fragrances. a day in the life of a linalool molecule: Chemical communication in a plant-pollinator system. part 1: Linalool biosynthesis in flowering plants. Plant Species Biol. 14, 95–120. doi: 10.1046/j.1442-1984.1999.00014.x

[B38] Reeves P. H. Ellis C. M. Ploense S. E. Wu M.-F. Yadav V. Tholl D.. (2012). A regulatory network for coordinated flower maturation. PloS Genet. 8, e1002506. doi: 10.1371/journal.pgen.1002506 22346763 PMC3276552

[B39] Roy R. Schmitt A. J. Thomas J. B. Carter C. J. (2017). Review: Nectar biology: From molecules to ecosystems. Plant Sci. 262, 148–164. doi: 10.1016/j.plantsci.2017.04.012 28716410

[B40] SAS Institute (2016). The SAS system for windows, version 9.4 (Cary NC, USA: SAS Institute).

[B41] Schmitt A. J. Roy R. Klinkenberg P. M. Jia M. Carter C. J. (2018). The octadecanoid pathway, but not COI1, is required for nectar secretion in *Arabidopsis thaliana* . Front. Plant Sci. 9. doi: 10.3389/fpls.2018.01060 PMC6092685 30135692

[B42] Schuppert G. F. Tang S. Slabaugh M. B. Knapp S. J. (2006). The sunflower high-oleic mutant ol carries variable tandem repeats of FAD2-1, a seed-specific oleoyl-phosphatidyl choline desaturase. Mol. Breed. 17, 241–256. doi: 10.1007/s11032-005-5680-y

[B43] Simpson B. B. Neff J. L. (1983). “Evolution and diversity of floral rewards,” in Handbook of experimental pollination biology (New York, NY, U.S.A.:Van Nostrand Reinhold Company Inc), pg. 142–159.

[B44] Talukder Z. I. Gong L. Hulke B. S. Pegadaraju V. Song Q. Schultz Q.. (2014). A high-density SNP map of sunflower derived from RAD-sequencing facilitating fine-mapping of the rust resistance gene R12. PloS One 9, e98628. doi: 10.1371/journal.pone.0098628 25014030 PMC4094432

[B45] Thomson J. D. Maddison W. P. Plowright R. C. (1982). Behavior of bumble bee pollinators of aralia hispida vent. (Araliaceae). Oecologia 54, 326–336. doi: 10.1007/BF00380001 28309956

[B46] Todesco M. Bercovich N. Kim A. Imerovski I. Owens G. L. Ruiz O. D.. (2022). Genetic basis and dual adaptive role of floral pigmentation in sunflowers. ELife 11, e72072. doi: 10.7554/eLife.72072 35040432 PMC8765750

[B47] Todesco M. Owens G. L. Bercovich N. Legare J.-S. Soudi S. Burge D. O.. (2020). Massive haplotypes underlie ecotypic differentiation in sunflowers. Nature 584, 602–607. doi: 10.1038/s41586-020-2467-6 32641831

[B48] United States Department of Agriculture [USDA] (2015). Attractiveness of agricultural crops to pollinating bees for the collection of nectar and/or pollen (Washington, DC: USDA).

[B49] van der Auwera G. A. Carneiro M. O. Hartl C. Poplin R. del Angel G. Levy-Moonshine A.. (2013). From FastQ data to high-confidence variant calls: The genome analysis toolkit best practices pipeline. Curr. Protoc. Bioinf. 43. doi: 10.1002/0471250953.bi1110s43 PMC4243306 25431634

[B50] van der Auwera G. O’Connor B. D. (2020). Genomics in the cloud: using docker, GATK, and WDL in terra. first edition (Sebastopol, CA: O’Reilly Media).

[B51] von Arx M. Goyret J. Davidowitz G. Raguso R. A. (2012). Floral humidity as a reliable sensory cue for profitability assessment by nectar-foraging hawkmoths. Proc. Natl. Acad. Sci. U.S.A. 109, 9471–9476. doi: 10.1073/pnas.1121624109 22645365 PMC3386090

[B52] Yang R. Yi N. Xu S. (2006). Box–cox transformation for QTL mapping. Genetica 128, 133–143. doi: 10.1007/s10709-005-5577-z 17028946

[B53] Zimmerman M. (1983). Plant reproduction and optimal foraging: Experimental nectar manipulations in delphinium nelsonii. Oikos 41, 57. doi: 10.2307/3544346

